# Adherence to and acceptability of three alcohol‐free, antiseptic oral rinses: A community‐based pilot randomized controlled trial among pregnant women in rural Nepal

**DOI:** 10.1111/cdoe.12562

**Published:** 2020-07-13

**Authors:** Daniel J. Erchick, Nitin K. Agrawal, Subarna K. Khatry, Joanne Katz, Steven C. LeClerq, Mark A. Reynolds, Luke C. Mullany

**Affiliations:** ^1^ Department of International Health Johns Hopkins Bloomberg School of Public Health Baltimore MD USA; ^2^ Department of Dentistry Institute of Medicine Tribhuhvan University Kathmandu Nepal; ^3^ Nepal Nutrition Intervention Project – Sarlahi (NNIPS) Kathmandu Nepal; ^4^ Department of Advanced Oral Sciences and Therapeutics University of Maryland School of Dentistry Baltimore MD USA

**Keywords:** gingivitis, oral health, oral hygiene, plaque control, public health

## Abstract

**Objectives:**

Antiseptic oral rinses have been evaluated as interventions to reduce the risk of adverse pregnancy outcomes associated with periodontal disease in pregnant women. Oral rinse use is not common in Nepal or other countries in South Asia, where the prevalence of adverse pregnancy outcomes is high. Understanding whether pregnant women in rural communities in this region would incorporate rinse use into their daily teeth cleaning routine is an important prerequisite to future research on this topic in South Asia.

**Methods:**

We conducted a community‐based pilot randomized controlled trial of three alcohol‐free, antiseptic oral rinses among pregnant women <22 weeks pregnant in rural Nepal with the aim of assessing rinse acceptability, adherence, and effect on clinical periodontal measures. At baseline, participants underwent a clinical periodontal examination, and then were classified as healthy or having at least mild gingivitis (≥1 site with probing depth (PD) 3 mm and bleeding on probing (BOP) or ≥4 mm (PD)). Participants were stratified by periodontal status and randomized within each exposure category to chlorhexidine (CHX) (0.12%), cetylpyridinium chloride (CPC) (0.05%), salt and water (NaCl), or control (no rinse). Rinse participants were followed weekly for 12 weeks, and all participants underwent a second periodontal examination and answered a questionnaire.

**Results:**

Pregnant women in the rural Terai region of Nepal showed high adherence to (mean weekly rinse use: 185 mL (standard deviation: 66 mL)) a recommended 210 mL and acceptability of all three rinses. Participants reported greater frequency of tooth brushing with toothpaste and improvements in other recommended oral hygiene behaviours. CHX significantly reduced rates of gingivitis (defined as a participant with BOP ≥ 10% of sites) and the extent of BOP (gingivitis at the end of follow‐up for CHX vs control: RR 0.37, 95% CI: 0.16, 0.84). CPC and NaCl rinse groups had rates of gingivitis and extent of BOP similar to the control group (gingivitis at the end of follow‐up for CPC: RR 0.81, 95% CI: 0.47, 1.38; NaCl: RR 0.92, 95% CI: 0.55, 1.56).

**Conclusions:**

Adherence to and acceptability of three alcohol‐free, antiseptic oral rinses were high among pregnant women in rural Nepal. Among participants with mild gingivitis at baseline, CHX rinse was most effective at reducing signs of disease compared to the control group. Oral rinse should be considered as a supplement to current oral self‐care routines for pregnant women in settings where rinse use is uncommon and access to oral health services is limited.

## 1 INTRODUCTION

Periodontal disease in pregnant women has been posited by some observational studies as a possible risk factor for adverse pregnancy outcomes, including preterm birth.^1^ Hypothesized mechanisms behind this association include haematogenic translocation of periodontal pathogens or their by‐products to the foetal‐placental unit, action of inflammatory mediators in the periodontium, or a confounding genetic hyper‐inflammatory phenotype.^2^, ^3^ Yet meta‐analyses of trials have not reported clear evidence of an effect of periodontal treatment vs no treatment on preterm birth (RR 0.87, 95% CI: 0.70, 1.10).^4^, ^5^, ^6^, ^7^ Further, a meta‐analysis of studies evaluating the efficacy of periodontal therapy to prevent preterm birth, low birth weight, and perinatal mortality, found evidence of publication bias for each of these outcomes.^8^

Systematic reviews have attributed inconsistent findings among trials to heterogeneity between studies, low methodological quality, and differences in when during pregnancy the intervention was delivered.^9^ One meta‐analysis, however, reported a significant reduction in risk of preterm birth in a sub‐group of five studies using a chlorhexidine rinse (CHX) as a co‐intervention with periodontal treatment (RR 0.69, 95% CI, 0.50, 0.95).^10^ In another review, among a sub‐group of four studies, a significant effect of treatment in reduction of preterm was found for women with high risk of the outcome (defined as incidence of preterm ≥15%) (RR 0.66, 95% CI: 0.54, 0.80).^7^

Antiseptic oral rinses are known to reduce gingival bleeding, inflammation, and the presence of aggressive periodontal pathogens associated with progression of periodontal disease, including *P gingivalis* and *A actinomycetemcomitans*.^11^, ^12^, ^13^, ^14^, ^15^ Studies have shown CHX to result in greater improvements in gingival index scores and other clinical measures than rinses with different agents, but this comes with a greater risk of side‐effects, such as tooth staining and transient taste loss; these do not commonly occur with other rinses, such as cetylpyridinium chloride (CPC).^16^ Salt and water rinse (NaCl), while not as effective as CHX or other agents, has shown some antimicrobial effect and would be a cheaper and easier option.^17^

An antimicrobial oral rinse, along with oral hygiene instruction, as an intervention for preterm birth and other adverse pregnancy outcomes would be appealing in many low‐ and middle‐income settings, where access to quality oral care is limited. Use of antiseptic oral rinses is not common in Nepal or other countries in South Asia.^18^, ^19^ It is unclear whether pregnant women in rural communities in this region would take up the practice of daily rinse use as part of their oral self‐care routine.

We conducted a community‐based randomized controlled trial of three alcohol‐free, antiseptic oral rinses, including CHX, CPC, and NaCl. Our primary aim was to assess levels of adherence to and acceptability of these three oral rinses over a 12‐week period among pregnant women in rural Sarlahi District, Nepal. A secondary aim of the trial was to evaluate changes in clinical periodontal measurements between examinations at baseline and the end of the 12‐week follow‐up period in both healthy participants and participants with signs of mild gingivitis by rinse assignment.

## 2 METHODS

We conducted a randomized controlled trial nested within the Nepal Oral Health Cohort Study (NOHCS), a community‐based, prospective cohort study of maternal gingivitis and adverse pregnancy outcomes in a sub‐area of Sarlahi District, Nepal, between January and November 2016. NOHCS participants were identified and determined eligible using the infrastructure of a large community‐based randomized trial, the Nepal Oil Massage Study (NOMS) (NCT01177111), which was actively enrolling a population‐based sample of pregnant women in this study area.

**FIGURE 1:**
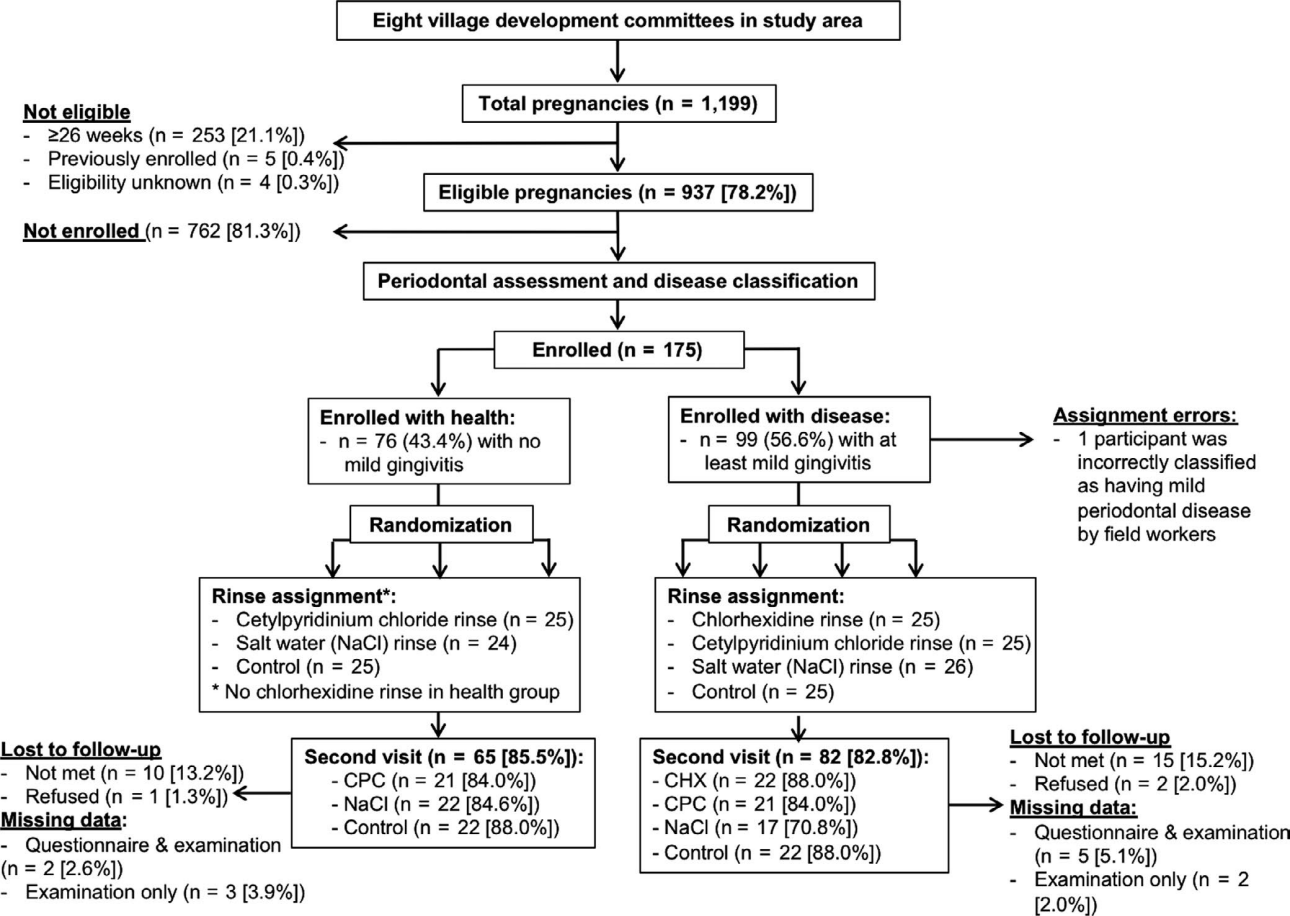
Participation flow chart

Pregnant women <22 weeks gestation from across eight village development committees (VDCs) in Sarlahi District were enrolled in the trial and followed for 12 weeks. A clinical periodontal examination conducted at the enrolment visit was used to assign participants into two groups – healthy and at least mild gingivitis. Within the healthy group, we randomly assigned 25 participants to one of two rinse groups (CPC, NaCl, and a control group). Within the disease group, we randomly assigned 25 participants to one of three rinse groups (CPC, NaCl, and CHX) or the control. CHX rinse was not used in healthy participants as this rinse is typically indicated as a therapeutic intervention only due to its strong side‐effects. In summary, group assignments by disease status were planned as the following: healthy (n = 25 CPC, n = 25 NaCl, n = 25 control) and disease (n = 25 CHX, n = 25 CPC, n = 25 NaCl, n = 25 control) for a total of 175 participants (Figure 1).

At the enrolment visit, all participants were consented for the trial, underwent a periodontal examination, were assigned to control or a rinse group, and received oral hygiene instructions, including a brushing demonstration. Those assigned to a rinse group also received their first batch of rinse at this time, and they were instructed not to dispose of the oral rinse containers after use.

After enrolment, participants in the three rinse groups were visited weekly for 12 weeks. Adherence was measured by observing the amount of rinse remaining in containers delivered the previous week and through questions about daily use in the previous 7 days. Participants were also asked basic questions on the acceptability of the rinse. Early exit from the trial occurred when participants either had the birth outcome (live birth, stillbirth, miscarriage or abortion) or refused further participation. At their 12th weekly visit, or at the time of early exit from the trial, all participants (both rinse and control groups) underwent a second oral health clinical examination. At this final visit, the women in the three rinse arms also answered a short questionnaire to gather additional information on oral rinse acceptability and adherence. Control participants were asked questions on oral hygiene behaviours during the 12‐week period.

All study visits, including oral health clinical examination, were performed in participant homes by five auxiliary nurse midwives who were trained by an experienced dentist (NKA) from the Department of Dentistry, Institute of Medicine, Tribhuvan University, Kathmandu, Nepal. The auxiliary nurse midwives conducted a full‐mouth periodontal examination, including measurement of PD at six sites per tooth (disto‐, mid‐, and mesial‐ aspects of buccal and lingual surfaces) and the CEJ‐GM distance on two sites per tooth (mid‐ buccal and lingual aspects), excluding third molars. After probing each quadrant, the auxiliary nurse midwives assessed presence or absence of BOP for buccal and lingual surfaces of each tooth. PD values were recorded in millimetres from 1 to 10, rounded to the next higher whole number. CEJ‐GM distances were recorded similarly, with values of 0 to 10 mm. If the free gingiva was coronal to the CEJ, the CEJ‐GM measurement was recorded as 0. Clinical attachment loss (CAL) was calculated by summing the PD and CEJ‐GM distance; the CEJ‐GM distance was assigned a value of 0 for distal and mesial sites, where this measure was not collected, and these sites were not considered in measures of CAL. We estimated the validity of PD measurements of the auxiliary nurse midwives relative to the dentist and found that per cent agreement, weighted kappa scores, and intraclass correlation coefficients, with an allowance of PD ± 1 mm, exceeded 99%, 0.7, and 0.9, respectively, indicating an acceptable level of agreement.^20^

A case definition of gingivitis was devised by our research team to allow the auxiliary nurse midwives to determine disease status (ie, healthy or at least mild gingivitis) in the participant's home directly after completion of the periodontal examination at the enrolment visit. We selected this approach for logistical reasons, primarily due to the large distances and travel times between participant homes in the study area. We defined healthy participants as those with no sites with probing depth (PD) 3 mm and bleeding on probing (BOP) or sites with PD ≥ 4 mm. Participants with at least mild gingivitis were defined as those with ≥1 site with PD 3 mm and BOP or ≥1 site with PD ≥ 4 mm.

During analysis, we applied a different definition for gingivitis, developed by the American Academy of Periodontology and European Federation of Periodontology. A case of clinical health was defined as a participant with all sites PD ≤ 3 mm and BOP < 10%.^21^, ^22^, ^23^ A case of clinical gingivitis was defined as a participant with BOP ≥ 10%, and further stratified as either localized gingivitis (BOP 10%‐30%) or generalized gingivitis (BOP ≥ 30%).^21^, ^22^

Specifications of the oral rinses were as follows: chlorhexidine gluconate salt 0.12% w/v., Clodine Mouthwash, Lomus Pharmaceuticals (Kathmandu, Nepal); cetylpyridinium chloride (0.05%), fluoride (0.05% w/w (225 ppmF), Active Total Care Sensitive Alcohol‐Free Mouthwash, Drammock International Ltd (Leeds, UK); and salt mouthwash, Lomus Pharmaceuticals (Kathmandu, Nepal).

Rinse participants were provided with a toothbrush and toothpaste and an initial amount of rinse, sufficient for 2 weeks, and small cup with a line marked at 15 mL. These participants were instructed to rinse twice daily with 15 mL of oral rinse, after brushing their teeth for 1 minute. Rinse safety information was also provided, including storage and how to keep the rinse away from children. Additional rinse was provided as needed by auxiliary nurse midwives during their weekly adherence visits to participant homes. Control participants received the same oral hygiene information as well as the toothbrush and toothpaste.

Baseline participant characteristics were compared between healthy and diseased groups across rinse assignment through bivariate analyses using cross‐tabulations and chi‐squared and Fisher's exact tests. We assessed differences in adherence and acceptability using chi‐squared tests and linear regression modelling as appropriate. Periodontal measures were analysed using an intention‐to‐treat approach. We calculated risk ratios for gingivitis (RR) at the end of follow‐up and associated 95% confidence internals (CI) using a log‐binomial regression models. All statistical analyses were performed in STATA 14.2 (StataCorp, College Station, TX, USA).

This study received ethical approved from the Institutional Review Board at Johns Hopkins Bloomberg School of Public Health (Baltimore, USA) and the Ethical Review Board of the Nepal Health Research Council (Kathmandu, Nepal).

## 3 RESULTS

A total of 175 pregnant women were enrolled in four groups of the pilot trial between June 27, 2016, and November 14, 2016. At the enrolment visit, one participant with gingivitis was incorrectly classified as healthy, a deviation from the protocol (described above), which yielded the following group assignments: healthy (n = 25 CPC, n = 24 NaCl, n = 25 control) and disease (n = 25 CHX, n = 25 CPC, n = 26 NaCl, n = 25 control).

Mean follow‐up time for rinse and control participants was 10.4 weeks (SD: 3.7 weeks), and total follow‐up time did not differ across rinse and control groups (*P* = .555). Nearly three‐quarters (n = 92/125, 73.6%) of rinse participants were met for at least 11 visits and over half (n = 69/125, 55.2%) were successfully followed for the full length of the trial (enrolment visit followed by 12 roughly weekly visits). During the weekly follow‐up visits, five (n = 5/175, 2.9%) participants refused rinse use for at least 1 week. At the end of follow‐up, for the second clinical examination visit, 25 (n = 25/175, 14.3%) participants were lost to follow‐up and three (n = 3/175, 1.7%) refused the visit. Due to a logistical error, data were lost from this visit (either questionnaire and examination data or only examination data) for 12 participants (n = 12/175, 6.9%).

Demographic and oral health characteristics of participants in the broader cohort study are described elsewhere.^18^ At baseline, within both the healthy or gingivitis groups, there were no significant differences in clinical periodontal measurements by rinse assignment. Characteristics of participants enrolled in the trial were balanced across intervention groups in the healthy and disease categories (Table 1).

**TABLE 1:** Participant characteristics (n = 175), by group (parentheses contain percentages)

Characteristic	All (n = 175)	Control	CHX	CPC	NACL	Incomplete follow‐up (n = 40)	Complete follow‐up (n = 135)^a^	*P*‐value^b^
Age (y)								
<20	51 (29.1)	14 (28.0)	3 (12.0)	16 (32.0)	18 (36.0)	15 (37.5)	36 (26.7)	.202
20‐24	77 (44.0)	24 (48.0)	14 (56.0)	23 (46.0)	16 (32.0)	16 (40.0)	61 (45.2)	
25‐29	36 (20.6)	10 (20.0)	5 (20.0)	7 (14.0)	14 (28.0)	6 (15.0)	30 (22.2)	
30‐34	8 (4.6)	2 (4.0)	2 (8.0)	4 (8.0)	0 (0.0)	1 (2.5)	7 (5.2)	
≥35	3 (1.7)	0 (0.0)	1 (4.0)	0 (0.0)	2 (4.0)	2 (5.0)	1 (0.7)	
Ethnic group								
Hills (Pahadi)	13 (7.4)	2 (4.0)	2 (8.0)	5 (10.0)	4 (8.0)	2 (5.0)	11 (8.2)	.505
Plains (Madeshi)	162 (92.6)	48 (96.0)	23 (92.0)	45 (90.0)	46 (92.0)	38 (95.0)	124 (91.9)	
BMI								
Underweight (<18.5 kg)	47 (26.9)	12 (24.0)	6 (24.0)	14 (28.0)	15 (30.0)	10 (25.0)	37 (27.4)	.630
Normal weight (18.5‐<25 kg)	119 (68.0)	35 (70.0)	18 (72.0)	33 (66.0)	33 (66.0)	29 (72.5)	90 (66.7)	
Overweight or obese (≥25 kg)	9 (5.1)	3 (6.0)	1 (4.0)	3 (6.0)	2 (4.0)	1 (2.5)	8 (5.9)	
Gravidity								
First pregnancy	55 (31.4)	17 (34.0)	3 (12.0)	14 (28.0)	21 (42.0)	15 (37.5)	40 (29.6)	.627
1‐3 pregnancies	104 (59.4)	29 (58.0)	21 (84.0)	29 (58.0)	25 (50.0)	22 (55.0)	82 (60.7)	
≥4 pregnancies	16 (9.1)	4 (8.0)	1 (4.0)	7 (14.0)	4 (8.0)	3 (7.5)	13 (9.6)	
Literacy								
No	97 (55.4)	27 (54.0)	17 (68.0)	26 (52.0)	27 (54.0)	23 (57.5)	74 (54.8)	.764
Yes	78 (44.6)	23 (46.0)	8 (32.0)	24 (48.0)	23 (46.0)	17 (42.5)	61 (45.2)	
Education (y)								
0	95 (54.3)	27 (54.0)	17 (68.0)	24 (48.0)	27 (54.0)	23 (57.5)	72 (53.3)	.898
1‐9	47 (26.9)	14 (28.0)	5 (20.0)	13 (26.0)	15 (30.0)	10 (25.0)	37 (27.4)	
≥10	33 (18.9)	9 (18.0)	3 (12.0)	13 (26.0)	8 (16.0)	7 (17.5)	26 (19.3)	
Electricity								
No	13 (7.4)	2 (4.0)	2 (8.0)	4 (8.0)	5 (10.0)	3 (7.5)	10 (7.4)	.984
Yes	162 (92.6)	48 (96.0)	23 (92.0)	46 (92.0)	45 (90.0)	37 (92.5)	125 (92.6)	
House construction material								
None, thatch, sticks or bamboo	109 (62.3)	33 (66.0)	18 (72.0)	29 (58.0)	29 (58.0)	27 (67.5)	82 (960.7)	.439
Wood planks, brick or stones & mortar	66 (37.7)	17 (34.0)	7 (28.0)	21 (42.0)	21 (42.0)	13 (32.5)	53 (39.3)	
House roof material								
None, plastic, thatch or grass	15 (8.6)	5 (10.0)	1 (4.0)	5 (10.0)	4 (8.0)	7 (17.5)	8 (5.9)	**.022**
Tile, tin or concrete	160 (91.4)	45 (90.0)	24 (96.0)	45 (90.0)	46 (92.0)	33 (82.5)	127 (94.1)	
Latrine								
No latrine	66 (37.7)	23 (46.0)	9 (36.0)	12 (24.0)	22 (44.0)	21 (52.5)	45 (33.3)	**.028**
Brick, concrete or pit latrine	109 (62.3)	27 (54.0)	16 (64.0)	38 (76.0)	28 (56.0)	19 (47.5)	90 (66.7)	

^a^ Complete follow‐up is defined as participants followed until the end of the study for whom the final questionnaire and second examination were administered and the data were available for analysis.

^b^ *P*‐value for chi‐square test of difference between complete data and incomplete data groups.

The total volume of rinse used by participants averaged 1921 mL (SD: 851 mL) of a recommended 2520 mL, ranging from 0 mL to 3700 mL (Table 2). Mean weekly rinse use (n = 123) was 185 mL (SD: 66 mL) of a recommended 210 mL. While few (n = 3/122, 2.5%) participants perfectly adhered to the twice per day rinse schedule (14 times per week), the mean number of rinses per week (n = 122) was 12.0 (SD: 2.7 times), and 91.8% (n = 112/122) reported rinsing at least 7 times per week.

**TABLE 2:** Adherence to oral rinse use instructions (n = 125), by group (parentheses contain percentages)

Measure	All	CHX	CPC	NaCl	*P*‐value^a^
Total rinse use (mL)^b^					
<1000	20 (16.0)	3 (12.0)	7 (14.0)	10 (20.0)	.595
1000‐1999	36 (28.8)	10 (40.0)	15 (30.0)	11 (22.0)	
2000‐2499	38 (30.4)	7 (28.0)	13 (26.0)	18 (36.0)	
≥2500	31 (24.8)	5 (20.0)	15 (30.0)	11 (22.0)	
Mean rinse use per week (mL)					
<100	6 (4.9)	0 (0.0)	3 (6.1)	3 (6.1)	.328
100‐149	33 (26.8)	11 (44.0)	12 (24.5)	10 (20.4)	
150‐199	34 (27.6)	5 (20.0)	16 (32.7)	13 (26.5)	
≥200	50 (40.7)	9 (36.0)	18 (36.7)	23 (46.9)	
Mean rinses per week					
<7	10 (8.2)	1 (4.0)	4 (8.2)	5 (10.4)	.885
7‐<10	5 (4.1)	2 (8.0)	1 (2.0)	2 (4.2)	
10‐13	43 (35.2)	9 (36.0)	18 (36.7)	16 (33.3)	
≥13	64 (52.5)	13 (52.0)	26 (53.1)	25 (52.1)	
Time of rinse use					
Morning	96 (100.0)	21 (100.0)	39 (100.0)	36 (100.0)	‐
Evening	90 (93.8)	20 (95.2)	36 (92.3)	34 (94.4)	.884
Afternoon	2 (2.1)	0 (0.0)	2 (5.1)	0 (0.0)	.225
Rinse use before teeth cleaning					
Never	89 (92.7)	17 (81.0)	36 (92.3)	36 (100.0)	**.030**
Sometimes	2 (2.1)	2 (9.5)	0 (0.0)	0 (0.0)	
Most times	5 (5.2)	2 (9.5)	3 (7.7)	0 (0.0)	
Rinse use after teeth cleaning					
Never	26 (27.1)	5 (23.8)	10 (25.6)	11 (30.6)	.101
Sometimes	6 (6.2)	4 (19.0)	1 (2.6)	1 (2.8)	
Most times	64 (66.7)	12 (57.1)	28 (71.8)	24 (66.7)	
Ate within 20 min of rinse use					
Never	90 (93.8)	19 (90.5)	37 (94.9)	34 (94.4)	.460
Sometimes	5 (5.2)	1 (4.8)	2 (5.1)	2 (5.6)	
Most times	1 (1.0)	1 (4.8)	0 (0.0)	0 (0.0)	
Drank within 20 min of rinse use					
Never	85 (88.5)	20 (95.2)	33 (84.6)	32 (88.9)	.257
Sometimes	9 (9.4)	0 (0.0)	6 (15.4)	3 (8.3)	
Most times	2 (2.1)	1 (4.8)	0 (0.0)	1 (2.8)	
Rinsed mouth with water within 20 min of rinse use					
Never	71 (74.0)	16 (76.2)	24 (61.5)	31 (86.1)	.055
Sometimes	6 (6.2)	1 (4.8)	2 (5.1)	3 (8.3)	
Most times	19 (19.8)	4 (19.0)	13 (33.3)	2 (5.6)	

^a^ Expected rinse use for the full 12‐wk trial was 2520 mL.

^b^ Chi‐squared test.

The mean volume of oral rinse used per session was 14.4 mL (SD: 1.7 mL). When directly observed by our data collectors, nearly all participants (n = 91/92, 98.9%) correctly used the rinse, including first cleaning their teeth, using the correct volume (15 mL), and rinsing for 1 minute. Instances of incorrect usage involved the wrong volume of rinse; for example, several participants (n = 10) used more than 15 mL because they had lost the measuring cap.

Rinse use in the morning and evening was most common, and adherence to rinse use instructions was high. Two (n = 2/96, 2.1%) participants reported ever swallowing the rinse. Few participants (n = 2/96, 2.1%) reported that a family member (husband) used their rinse, but more (n = 17/96, 17.7%) reported that it was a challenge to keep the rinse away from their children. Rinse adherence was similar between rinse groups, with one exception; four participants in the chlorhexidine group reported sometimes using the rinse before cleaning their teeth.

Nearly all participants in each group reported liking the rinse to some degree when questioned at the end of the trial (Table 3). Responding about the individual characteristics of the rinse, taste or smell were the two most common aspects of the rinse that participants reported disliking. We found no significant differences in rinse acceptability between rinse groups for any of these questions.

**TABLE 3:** Acceptability of oral rinses, by group (parentheses contain percentages)

Measure	All	CHX	CPC	NACL	*P*‐value^a^
Overall opinion of rinse					
Did not like	3 (3.1)	1 (4.8)	1 (2.6)	1 (2.8)	.846
Liked a little	30 (31.2)	7 (33.3)	10 (25.6)	13 (36.1)	
Liked moderately	31 (32.3)	6 (28.6)	16 (41.0)	9 (25.0)	
Liked significantly	32 (33.3)	7 (33.3)	12 (30.8)	13 (36.1)	
Opinion of rinse taste					
Dislike	13 (13.5)	2 (9.5)	5 (12.8)	6 (16.7)	.738
Like	83 (86.5)	19 (90.5)	34 (87.2)	30 (83.3)	
Opinion of rinse smell					
Dislike	14 (14.6)	5 (23.8)	5 (12.8)	4 (11.1)	.390
Like	82 (85.4)	16 (76.2)	34 (87.2)	32 (88.9)	
Opinion of rinse colour					
Dislike	2 (2.1)	1 (4.8)	0 (0.0)	1 (2.8)	.437
Like	94 (97.9)	20 (95.2)	39 (100.0)	35 (97.2)	
Opinion of rinse presentation					
Dislike	1 (1.0)	1 (4.8)	0 (0.0)	0 (0.0)	.165
Like	95 (99.0)	20 (95.2)	39 (100.0)	36 (100.0)	
How mouth felt using rinse					
Less clean	27 (28.1)	7 (33.3)	11 (28.2)	9 (25.0)	.875
Same	10 (10.4)	3 (14.3)	4 (10.3)	3 (8.3)	
More clean	59 (61.5)	11 (52.4)	24 (61.5)	24 (66.7)	
Would use again if pregnant					
No	1 (1.0)	0 (0.0)	1 (2.6)	0 (0.0)	.478
Yes	95 (99.0)	21 (100.0)	38 (97.4)	36 (100.0)	
Would purchase rinse					
No	21 (22.1)	2 (10.0)	13 (33.3)	6 (16.7)	.075
Yes	74 (77.9)	18 (90.0)	26 (66.7)	30 (83.3)	

^a^ Chi‐squared test.

Most participants (n = 88/96 (91.7%) in the three rinse groups reported experiencing positive changes associated with use of the rinse. Participants saw a decrease in plaque (n = 51/96, 53.1%) gum redness (n = 31/96, 32.3%), bleeding (n = 25/96, 26.0%), foul breath (n = 23/96, 24.0%), and pain (n = 16/96, 16.7%). There were no significant differences in self‐reported effects of the rinse among oral rinse groups. Some participants (n = 8/96, 8.3%) reported negative changes associated with use of the rinse. Two participants (n = 2/96, 2.1%), one each from the CHX and CPC groups, noticed staining of their teeth. A few participants in the NaCl group reported an increase in gum redness (n = 3/96, 3.1%), bleeding (n = 3/96, 3.1%), or foul breath (n = 1/96, 1.0%) during this period.

At the end of trial, participants reported an average increase in teeth cleaning of 2.2 times per week (95% CI: 1.2, 3.2), relative to behaviours before enrolment in the study. Additionally, the proportion of participants having cleaned their teeth at least twice the day prior increased from 41.3% to 76.9% (*P* < .001). Use of a toothbrush increased from 72.8% to 97.8% (*P* < .001), while neem and bamboo datiwan (दतिवन, a local teeth cleaning instrument fashioned from twigs of a variety of trees) use decreased from 8.7% to 2.8% (*P* = .04) and 31.4% to 2.2% (*P* < .001), respectively. Use of only fingers for teeth cleaning decreased from 14.0% to 0.7% (*P* < .001). Toothpaste use increased from 57.0% to 98.6% (*P* < .001) and dant manjan (दांत मंजन, a common powder for teeth cleaning) use decreased 18.6% to 1.4% (*P* < .001). Those initially using sand (n = 3), charcoal (n = 2), and ash (n = 1) stopped doing so.

Among the healthy group at the end of follow‐up, CPC participants had similar proportions of gingivitis and extent of BOP to the control group; NaCl participants had a higher proportion of participants with ≥1 site BOP than the control group (RR 1.56, 95% CI: 1.02, 2.38), although this difference was not observed (RR 1.37, 95% CI: 0.43, 4.42) for our primary definition of gingivitis (Tables 4 and 5).

**TABLE 4:** Periodontal measurements among women with health at baseline and the end of the study, by group (parentheses contain percentages or standard deviation)

Measure	Baseline	End of follow‐up				
	All (n = 76)	All (n = 60)	Control	CPC	NACL	*P*‐value^a^
Initial disease classification						
0 sites PD = 3 mm & BOP or PD ≥ 4 mm	76 (100.0)	39 (65.0)	17 (77.3)	14 (77.8)	8 (40.0)	**.016**
≥1 site PD = 3 mm & BOP or PD ≥ 4 mm	0 (0.0)	21 (35.0)	5 (22.7)	4 (22.2)	12 (60.0)	
Clinical health and gingivitis						
Health (All sites PD ≤ 3 mm & BOP < 10%)	66 (86.8)	45 (75.0)	18 (81.8)	12 (66.7)	15 (75.0)	.545
Gingivitis (BOP ≥ 10% and/or PD ≥ 4 mm)	10 (13.2)	15 (25.0)	4 (18.2)	6 (33.3)	5 (25.0)	
‐ Localized gingivitis (BOP < 30%)	10 (100.0)	15 (100.0)	4 (100.0)	6 (100.0)	5 (100.0)	N/A
‐ Generalized gingivitis (BOP ≥ 30%)	0 (0.0)	0 (0.0)	0 (0.0)	0 (0.0)	0 (0.0)	
Bleeding on probing (BOP)						
Per cent of sites BOP (mean ± SD)	4.5 ± 5.2	6.2 ± 7.4	5.2 ± 7.7	6.5 ± 8.2	6.9 ± 6.4	N/A
No sites BOP	25 (32.9)	19 (31.7)	10 (45.5)	6 (33.3)	3 (15.0)	.104
≥1 site BOP	51 (67.1)	41 (68.3)	12 (54.5)	12 (66.7)	17 (85.0)	
≥1 site BOP & BOP < 10%	41 (54.0)	26 (43.3)	8 (36.4)	6 (33.3)	12 (60.0)	.203
BOP ≥ 10% & BOP < 30%	10 (13.2)	15 (25.0)	4 (18.2)	6 (33.3)	5 (25.0)	
BOP ≥ 30%	0 (0.0)	0 (0.0)	0 (0.0)	0 (0.0)	0 (0.0)	
Probing depth (PD)						
Mean PD (mm) (mean ± SD)	1.6 ± 0.2	1.6 ± 0.2	1.6 ± 0.2	1.6 ± 0.2	1.6 ± 0.2	N/A
≥1 site PD ≥ 4 mm	0 (0.0)	1 (1.7)	1 (4.6)	0 (0.0)	0 (0.0)	.415
Clinical attachment loss (CAL)						
Mean CAL direct sites (mm) (mean ± SD)	1.6 ± 0.2	1.6 ± 0.2	1.6 ± 0.2	1.6 ± 0.2	1.6 ± 0.2	N/A
≥1 site recession ≥ 1 mm	3 (4.0)	2 (3.3)	0 (0.0)	0 (0.0)	2 (10.0)	.126
≥1 site CAL ≥ 4 mm	1 (1.3)	2 (3.3)	1 (4.6)	0 (0.0)	1 (5.0)	.640

^a^ *P*‐value for chi‐square test of difference between four groups (three rinse and control) at the end of follow‐up visit.

**TABLE 5:** Periodontal measurements among women with gingivitis at baseline and the end of the study, by group (parentheses contain percentages or standard deviation)

Measure	Baseline	End of follow‐up					
	All (n = 99)	All (n = 75)	Control	CHX	CPC	NACL	*P*‐value^a^
Initial disease classification							
0 sites PD = 3 mm & BOP or PD ≥ 4 mm	0 (0.0)	26 (34.7)	4 (20.0)	13 (61.9)	6 (31.6)	3 (20.0)	**.016**
≥1 site PD = 3 mm & BOP or PD ≥ 4 mm	99 (100.0)	49 (65.3)	16 (80.0)	8 (38.1)	13 (68.4)	12 (80.0)	
Clinical health and gingivitis							
Health (All sites PD ≤ 3 mm & BOP < 10%)	33 (33.3)	38 (50.7)	7 (35.0)	16 (76.2)	9 (47.4)	6 (40.0)	**.042**
Gingivitis (BOP ≥ 10% and/or PD ≥ 4 mm)	66 (66.7)	37 (49.3)	13 (65.0)	5 (23.8)	10 (52.6)	9 (60.0)	
‐ Localized gingivitis (BOP < 30%)	51 (77.3)	26 (70.3)	8 (61.5)	4 (80.0)	8 (80.0)	6 (66.7)	.751
‐ Generalized gingivitis (BOP ≥ 30%)	15 (22.7)	11 (29.7)	5 (38.5)	1 (20.0)	2 (20.0)	3 (33.3)	
Bleeding on probing (BOP)							
Per cent of sites BOP (mean ± SD)	18.2 ± 15.1	13.3 ± 15.1	18.1 ± 15.5	5.5 ± 8.6	15.1 ± 15.1	15.3 ± 18.7	N/A
No sites BOP	0 (0.0)	16 (21.3)	1 (5.0)	9 (42.9)	3 (15.8)	3 (20.0)	**.025**
≥1 site BOP	99 (100.0)	59 (78.7)	19 (95.0)	12 (57.1)	16 (84.2)	12 (80.0)	
≥1 site BOP & BOP < 10%	36 (36.4)	26 (34.7)	7 (35.0)	9 (42.9)	6 (31.6)	4 (26.7)	.075
BOP ≥ 10% & BOP < 30%	48 (48.5)	22 (29.3)	7 (35.0)	2 (9.5)	8 (42.1)	5 (33.3)	
BOP ≥ 30%	15 (15.2)	11 (14.7)	5 (25.0)	1 (4.8)	2 (10.5)	3 (20.0)	
Probing depth (PD)							
Mean PD (mm) (mean ± SD)	1.8 ± 0.3	1.7 ± 0.3	1.8 ± 0.3	1.7 ± 0.3	1.7 ± 0.2	1.8 ± 0.3	N/A
≥1 site PD ≥ 4 mm	16 (16.2)	9 (12.0)	5 (25.0)	2 (9.5)	0 (0.0)	2 (13.3)	.115
Clinical attachment loss (CAL)							
Mean CAL direct sites (mm) (mean ± SD)	1.8 ± 0.3	1.7 ± 0.3	1.9 ± 0.3	1.7 ± 0.3	1.7 ± 0.2	1.8 ± 0.3	N/A
≥1 site recession ≥ 1 mm	16 (16.2)	11 (14.7)	4 (20.0)	4 (19.1)	0 (0.0)	3 (20.0)	.223
≥1 site CAL ≥ 4 mm	23 (23.2)	13 (17.3)	6 (30.0)	3 (14.3)	0 (0.0)	4 (26.7)	.640

^a^ *P*‐value for chi‐square test of difference between four groups (three rinse and control) at the end of follow‐up visit.

Among the disease group at the end of follow‐up, gingivitis was 63% lower for CHX participants vs control (RR 0.37, 95% CI: 0.16, 0.84) (Table 6). CPC participants had a slightly lower proportion of gingivitis and extent of BOP than the control group, but these differences were not statistically significant. NaCl participants were statistically similar to the control group, although NaCl group showed a nonstatistically significant difference in extent of BOP from the control (Control 5% no BOP vs NaCl 20% no BOP).

**TABLE 6:** Risk ratios for gingivitis at the end of the study, by rinse group

Measure	Health (n = 60)	Disease (n = 75)
Gingivitis at the end of follow‐up (RR, 95% CI)^a^		
Control	Ref	Ref
CHX	‐	**0.37 (0.16, 0.84)**
CPC	1.83 (0.61, 5.51)	0.81 (0.47, 1.38)
NaCl	1.37 (0.43, 4.42)	0.92 (0.55, 1.56)

^a^ Risk ratio of gingivitis at the end of the study for rinse group vs control and 95% CI.

There were no statistically significant differences in mean PD, proportion of sites with PD ≥ 4 mm, or clinical attachment loss (CAL) by rinse assignment within healthy or disease groups at the end of follow‐up.

## 4 DISCUSSION

We conducted a pilot community‐based randomized controlled trial to understand the adherence to and acceptability of three nonalcohol, antiseptic oral rinses among young pregnant women in rural Nepal. Generally, across the three rinses, we observed high adherence to rinse protocol, and participants reported positive opinions of the rinse taste, smell, and other characteristics. These findings suggest that pregnant women might be willing to incorporate an oral rinse into their daily oral hygiene routine.

Oral rinse use is uncommon in Nepal and other countries in South Asia. A survey of women in this study population that found the prevalence of both dental floss and oral rinse use to be <1%.^18^ Another study, conducted in a predominantly urban and semi‐urban area of Dharwad District in Karnataka, India, reported a prevalence of oral rinse use of 1% among Indian adults.^19^ Although rinse use in this region is uncommon, our findings are supported by a qualitative study conducted by our study team that reported that women in this population would be willing to use an oral rinse during pregnancy if it might have a positive impact on the baby's health; this could also explain the high percentage of women who reported being willing to use an oral rince if they became pregnant again.^24^

We additionally considered several secondary outcomes, including changes in oral hygiene behaviours and clinical periodontal measurements. Of three rinses, only CHX significantly improved clinical periodontal measurements. Improvements in the CPC group were smaller and may have been statistically significant with a larger sample size. A greater benefit from CPC use may also have been observed in a study population with more severe periodontal disease. Among control participants, we did not observe worsening of clinical periodontal measurements over time that would be expected from the normal effect of hormonal changes during pregnancy—perhaps a positive result of improved oral hygiene behaviours reported by participants in this study.^25^

Antiseptic oral rinses have been considered for their potential as interventions to reduce risk of adverse pregnancy outcomes through treatment of periodontal disease. This is of particular interest because few effective interventions to prevent preterm birth exist, and therapeutic interventions to help preterm babies survive are difficult to scale up in South Asia, where many women deliver at home without skilled care and rates of preterm are high (14% in Nepal in 2010).^26^, ^27^ A review by Boutin et al (2013), in a meta‐analysis of twelve randomized controlled trials, found a significant reduction in risk of preterm birth (<37 weeks) (RR 0.69, 95% CI: 0.50, 0.95) in a subset of five studies where CHX oral rinse was provided as part of the intervention.^10^, ^28^, ^29^, ^30^, ^31^, ^32^ The review also reported a significant reduction in risk of low birth weight in a meta‐analysis of four studies (RR 0.44, 95% CI: 0.31, 0.65).^10^ While most previous studies of this relationship targeted women with periodontitis, one of the trials in this review, by Lopez et al (2005), intervened on women with gingivitis in a population in Chile, reporting reduced risk of preterm low birth weight.^29^

Jeffcoat et al (2011) found a reduction in incidence of preterm birth (<35 weeks, RR 0.26, 95% CI: 0.10, 0.70) and low birth weight with the use of nonalcohol CPC oral rinse intervention, oral hygiene instructions and home care supplies (toothbrushes, fluoride toothpaste, and dental floss) in a population of high‐risk women in the United States.^33^ However, Jiang et al (2016), in a study administering a CPC oral rinse and oral hygiene education intervention to a population of women in rural China, reported improved periodontal health but no change in the rate of preterm birth (OR 1.59, 95% CI: 0.51, 4.92) or low birth weight, although they did observe a reduction in risk for premature rupture of membranes (OR 0.23, 95% CI: 0.07, 0.84).^34^ Jiang et al (2016) proposed differences in their study and that of Jeffcoat et al (2011) that could be responsible for these results, including that participants in the Jeffcoat et al study were at high‐risk of preterm birth, while their participants were from a population at low‐risk.^34^

Some have argued that periodontal therapy may not fully disrupt the causal pathway between periodontal disease and preterm birth.^35^ Xiong el al. (2011) argued that bacteraemia and elevated inflammation caused by the mechanical manipulation of the gingiva involved in periodontal therapy could contribute to increased risk of adverse pregnancy outcomes.^9^ Such a phenomenon could be responsible for the contradictory results between observational and interventional studies of this relationship. An oral rinse, as a single intervention or co‐intervention with root scaling and planning, could act more directly on this causal pathway by reducing inflammation, plaque biofilm and aggressive pathogens, and subsequent risk of haematogenic translocation of periodontal pathogens or by‐products to the foetal‐placental unit. This approach would be particularly suited to LMICs where there is little access to preventive care and nonsurgical procedures could be conducted by dental hygienists after appropriate training.

Limitations of this study included participant drop out, missing data points and a small sample size. A larger sample size could have allowed for comparison of birth outcomes between rinse and control groups. Logistical restraints prevented us from assigning participants to healthy and gingivitis groups using our primary exposure definition; in the future, using tablet computer or mobile applications for data collection could eliminate this problem, by allowing for more complicated clinical definitions to be utilized in real time. Lastly, although participants were <22 weeks gestation at enrolment, they ranged from 7 to 24 weeks (one participant was enrolled >22 weeks (24 weeks) due to a calculation error). Providing participants with an antiseptic rinse earlier in pregnancy, that is, restricted to only the first trimester, could potentially further reduce gingivitis and extent of bleeding during the course pregnancy in this population.

## 5 CONCLUSION

Our study demonstrated that adherence to and acceptability of three alcohol‐free, antiseptic oral rinses were high among pregnant women in rural Nepal. Among participants with mild gingivitis at baseline, CHX rinse was most effective at reducing signs of disease compared with the control group. Oral rinse should be considered as a supplement to current oral self‐care routines for pregnant women in settings where rinse use is uncommon and access to oral health services is limited.

## CONFLICT OF INTEREST

The authors declare that they have no competing interests.

## AUTHOR CONTRIBUTIONS

Dr Erchick conceptualized and designed the study, developed field implementation protocols, led data collection in the field, conducted the analysis and wrote the manuscript. Dr Agrawal conceptualized and designed the study, trained and oversaw the data collectors and provided comments on the manuscript. Dr Khatry conceptualized and designed the study, oversaw field implementation and ensured quality data collection and provided comments on the manuscript. Dr Katz conceptualized and designed the study, ensured quality data collection, advised on analytic approach and provided comments on the manuscript. Mr LeClerq conceptualized and designed the study, supported overall implementation in the field and provided comments on the manuscript. Dr Reynolds conceptualized and designed the study, contributed to the analysis and interpretation of the results and provided comments on the manuscript. Dr Mullany conceptualized and designed the study, obtained funding for the study, oversaw implementation of data collection, obtained ethical approvals, advised on analytic approach and provided edits and comments on the manuscript. All authors approved the final manuscript as submitted and agree to be accountable for all aspects of the work.
